# Genomic Characteristics of Genetic Creutzfeldt-Jakob Disease Patients with V180I Mutation and Associations with Other Neurodegenerative Disorders

**DOI:** 10.1371/journal.pone.0157540

**Published:** 2016-06-24

**Authors:** Sol Moe Lee, Myungguen Chung, Jae Wook Hyeon, Seok Won Jeong, Young Ran Ju, Heebal Kim, Jeongmin Lee, SangYun Kim, Seong Soo A. An, Sung Beom Cho, Yeong Seon Lee, Su Yeon Kim

**Affiliations:** 1 Division of Zoonoses, Center for Immunology & Pathology, National Institute of Health, Korea Centers for Disease Control & Prevention, Cheongju-si, Chungcheongbuk-do, South Korea; 2 Department of Agricultural Biotechnology, Animal Biotechnology Major, Seoul National University, Seoul, South Korea; 3 Division of Bio-Medical Informatics, Center for Genome Science, National Institute of Health, Korea Centers for Disease Control & Prevention, Cheongju-si, Chungcheongbuk-do, South Korea; 4 Division of Molecular and Life science, Hanyang University, Seoul, South Korea; 5 Department of Neurology, Seoul National University Bundang Hospital and Seoul National University College of Medicine, Gyeonggi-do, South Korea; 6 Gachon BioNano Research Institute, Gachon University, Gyeonggi-do, South Korea; Scuola Internazionale Superiore di Studi Avanzati, ITALY

## Abstract

Inherited prion diseases (IPDs), including genetic Creutzfeldt-Jakob disease (gCJD), account for 10–15% of cases of prion diseases and are associated with several pathogenic mutations, including P102L, V180I, and E200K, in the prion protein gene (*PRNP*). The valine to isoleucine substitution at codon 180 (V180I) of *PRNP* is the most common pathogenic mutation causing gCJD in East Asian patients. In this study, we conducted follow-up analyses to identify candidate factors and their associations with disease onset. Whole-genome sequencing (WGS) data of five gCJD patients with V180I mutation and 145 healthy individuals were used to identify genomic differences. A total of 18,648,850 candidate variants were observed in only the patient group, 29 of them were validated as variants. Four of these validated variants were nonsense mutations, six were observed in genes directly or indirectly related to neurodegenerative disorders (NDs), such as *LPA*, *LRRK2*, and *FGF20*. More than half of validated variants were categorized in Gene Ontology (GO) terms of binding and/or catalytic activity. Moreover, we found differential genome variants in gCJD patients with V180I mutation, including one uniquely surviving 10 years after diagnosis of the disease. Elucidation of the relationships between gCJD and Alzheimer’s disease or Parkinson’s disease at the genomic level will facilitate further advances in our understanding of the specific mechanisms mediating the pathogenesis of NDs and gold standard therapies for NDs.

## Introduction

Prion diseases (PrDs), also called transmissible spongiform encephalopathies (TSEs), are rare fatal neurodegenerative disorders (NDs) that affect both humans and animals. PrDs are characterized at the pathological level by abnormal accumulation of misfolding prion protein (PrP^Sc^), affecting the central nervous system (CNS) [1, 2]. In humans, the causative agent is encoded by the prion protein (*PRNP*) gene, found on chromosome 20p13. Several pathogenic mutations in *PRNP*, including P102L, P105L, D178N, V180I, E200K, and V203I, have been reported [3–11]. Pathogenic mutations causing inherited PrDs (IPDs), including genetic Creutzfeldt-Jakob disease (gCJD), fatal familial insomnia (FFI), and Gerstmann-Straussler-Scheinker syndrome (GSS), are responsible for 10–15% of all human PrDs cases [12, 13].

The incubation period for PrDs is influenced by mutations in *PRNP* [14, 15]. Transmission of PrP^Sc^ and the effects of mutations in *PRNP* have been proven in animal models, including primates and rodents [16–18]. In addition to *PRNP*, other genes (loci) are thought to be related to the incubation period of PrDs because of the incomplete penetrance of PrDs [19–23].

PrDs and other NDs, such as Alzheimer's disease (AD) and Parkinson’s disease (PD), share several clinical characteristics, including neuropathological features [24–27]. Moreover, in all of these disorders, abnormal accumulation of causative agents, such as prion protein, has been detected in the brains of affected individuals. For example, aggregates of tau and α-synuclein (SNCA) protein have been observed in the brains of patients with AD and PD, respectively [28–30]. Interestingly, SNCA aggregates have also been detected in PrDs-affected brains in sheep and goats [31].

The V180I mutation in *PRNP*, which is the most common cause of gCJD in East Asian patients, was reported first in 1993, and only a few cases of the V180I mutation have been reported in Europe [8, 32, 33]. Most cases of gCJD exhibit onset of symptoms in the late 70s, with the disease lasting 2–3 years [34]. A family history of gCJD with V180I mutation is infrequent, and the mutation has been observed in three non CJD individuals; thus, it is unclear whether this mutation causes PrDs [34, 35]. However, gCJD patients with V180I mutation show several clinical features unique from those of patients with sporadic CJD (sCJD) or gCJD with other mutations, such as less frequent myoclonus, cerebellar signs, and visual dysfunctions and slow progression [34, 36]. Thus, V180I is thought to be a pathogenic mutation in patients with gCJD, but may have low penetrance. Moreover, because of this incomplete penetrance, unknown genes may be involved in the pathogenesis of this disease. Thus, analysis of the relationships between gCJD and other NDs, such as AD and PD, may provide insights into unidentified genes affecting PrDs.

Therefore, in the current study, we aimed to identify genomic differences between healthy individuals and patients with PrDs, particularly gCJD patients with V180I mutation, and improve our understanding of the genetic relationships between other NDs and gCJD by analysis of genetic variants in gCJD patients with V180I mutation.

## Results

### Patient characteristics

Disease onset occurred between 72 and 77 years of age in four of the patients and at 57 years of age in one of the patients (Table 1). Patient no. 4, who experienced disease onset at 57 years of age, was diagnosed with gCJD in 2004. However, the patient visited the hospital again 8 years later (in 2012) and was still alive, although bedridden (last confirmed on October 13, 2015). With the exception of this patient, who showed atypical disease onset, the other patients in this study showed disease onset similar to those of other gCJD patients with V180I mutation [36, 37].

**Table 1:** Clinical features of the five gCJD patients with V180I mutation.

Patient no.	Diseases onset (years)	Disease progression	Clinical features
1	77	Rapid	Symptoms: Rapid progressive cognitive impairment, ataxia, dementia, and gait disturbance
			EEG: Diffuse slow wave
2	72	Rapid	Symptoms: Rapid progressive cognitive decline, depression
			MRI: No abnormal enhancing focal lesion in the brain
			DWI: High signal intensity in cortex of temporoparietal lobe, left frontal lobe, and left insular cortex
3	73	Rapid	Symptoms: Rapid progressive cognitive impairment
			EEG: Spike in right side frontal lobe
4	57	Slow	Symptoms: Muscle cramping, bedridden state
			EEG: Diffuse cerebral dysfunction
			MRI: T2 High signal intensity in superficial cortex and basal ganglia
5	73	-	Symptoms: Encephalopathy symptoms, stammering, and nausea continuously

Abbreviations: MRI, magnetic resonance imaging; DWI, diffusion weighted image; EEG, electroencephalogram

All five gCJD patients with V180I mutation were positive or weakly positive for 14-3-3 protein in the cerebral spinal fluid (CSF) (Table 2). In three of the patients, total tau (t-tau) protein titers were greater than the recommended cut-off level (1,000 pg/mL) [38]. The phospho-tau/total tau ratio (p/t tau ratio) have been recommended as second screening tau test, and recommended p/t tau ratio in ref. [38] were below than 4 × 10^−2^. The p/t tau ratio in the CSF of the four patients ranged from 2.209 × 10^−2^ to 10.093 × 10^−2^. Therefore, according to recommended tau protein detection criteria, patients no. 1 and 3 were within the range established for CJD.

**Table 2:** Results of biochemical analysis of the five gCJD patients with V180I.

Patient no.	14-3-3	t-tau (pg/mL)	p-tau (pg/mL)	p/t ratio	RT-QuIC with substrate replacement
1	Positive	**3088.005**	79.403	**0.02571**	Positive
2	Positive	780.45	17.24	**0.02209**	Positive
3	Positive	**1136.5**	28.2	**0.02481**	Positive
4	Weakly positive	-	-	-	-
5	Positive	**4537.8**	458	0.10093	Negative

Abbreviation: RT-QuIC, Real-time quaking induced conversion. 14-3-3 protein, tau protein detection and RT-QuIC with substrate replacement were performed using CSF. Scores within the range of CJD are displayed in bold.

**Fig 1:**
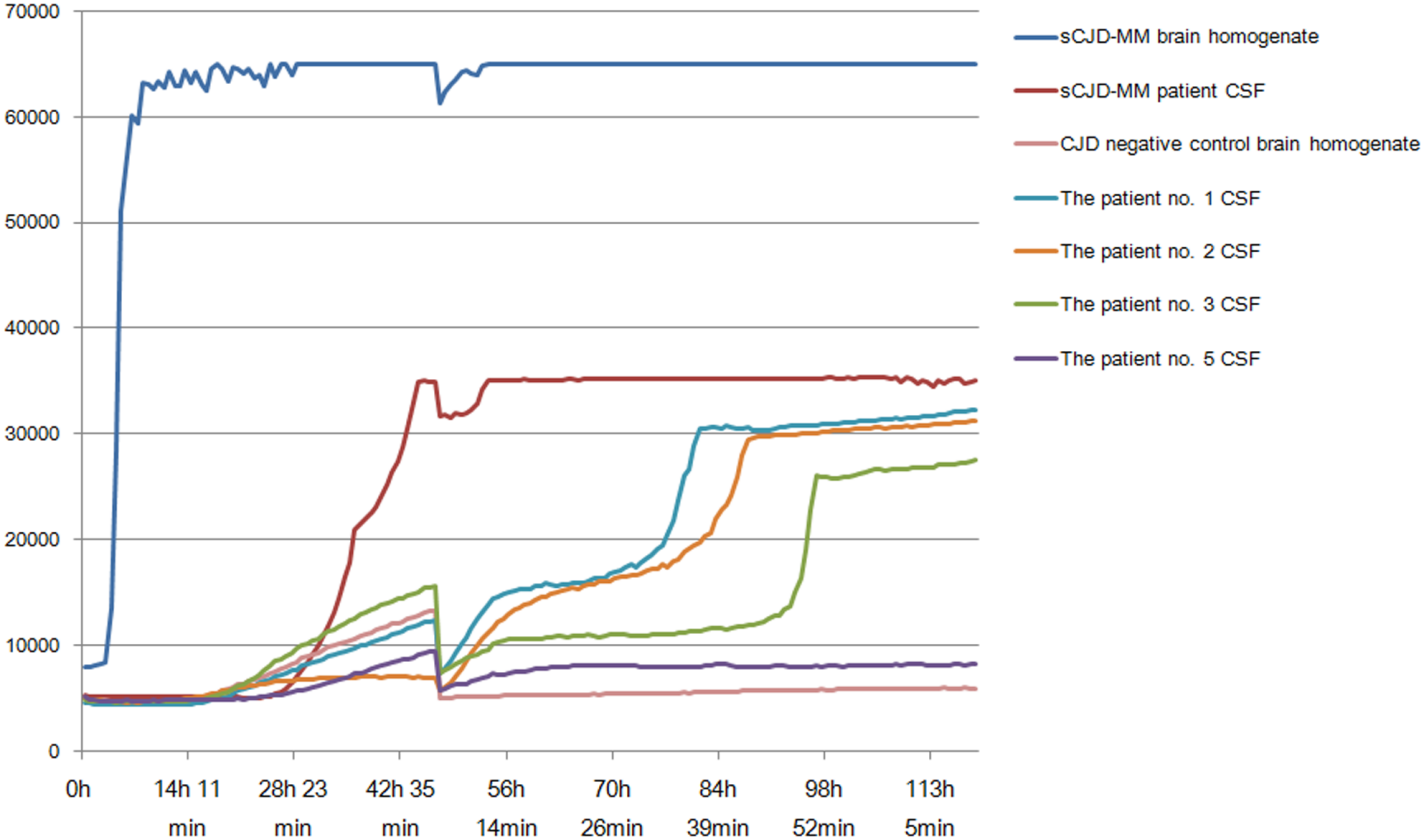
Results of RT-QuIC analysis. ThT fluorescence was measured by relative fluorescence units (rfu) with saturation occurring at 65,000 for every 42 min. The RT-QuIC responses were performed using 10^−3^ dilution of sCJD patient with 129MM type brain homogenate (NHBX0/0001), 10^−5^ CJD negative control brain homogenate (NHBZ0/0005), 15ul of 10^−1^ dilution of CSF from 4 gCJD cases with V180I (the Patients nos. 1, 2, 3 and 5) and 1 sCJD case with 129MM. Each point represents the mean of 4 replicate rfu readings.

Because the CSF of patient no. 4 was not stored, the RT-QuIC assay with substrate replacement was performed using CSF samples from only four patients, and the sensitivities were 75% (Fig 1).

### Whole genome data analysis

Raw data for whole genome information are shown in S1 and S2 Tables. A total 72–114 Gbp with Q30 of sequences was generated. The average read length was 126 bp, and about 51–92 Gbp were aligned with mapping quality of Q20 to the reference genome (hg19).

**Fig 2:**
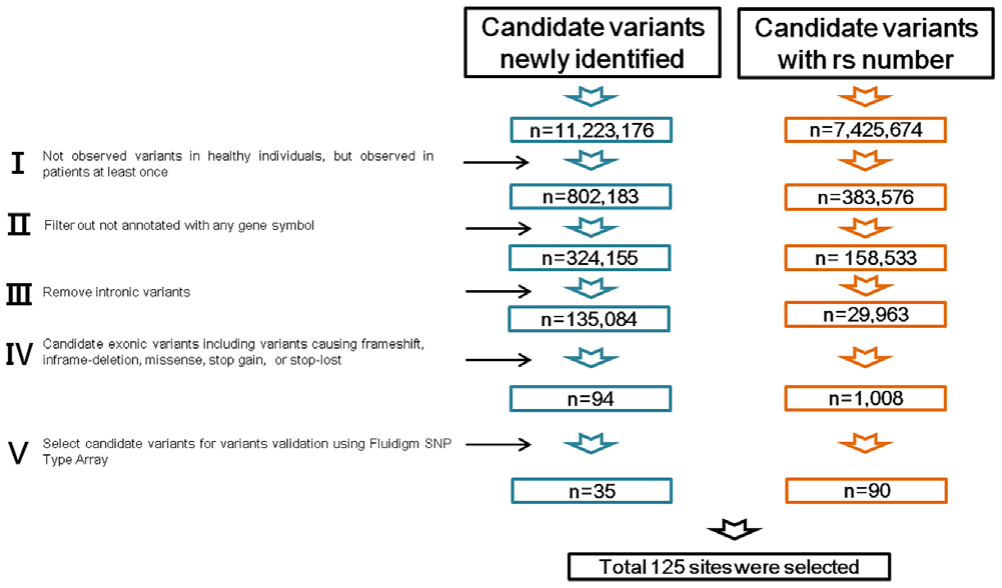
Variant filtering strategy of whole genome sequencing data. Overview of variant filtering to identify gCJD-related genes and gene variants. Each stage was processed followed by stage I–V.

The genomic variants in the five gCJD patients with V180I mutation were compared with 134 of 135 healthy individuals (see materials and methods, S3 Table and S1 File), and 125 of 18,648,850 variants were selected for validation (Fig 2). Of these 125 candidates, 76 were observed in all five patients, one was observed in four patients, two were observed in two patients, and 46 were observed in only one patient (S4 Table). From these candidates, to eliminate false positives, we performed validation of the 125 candidate variants using Fluidigm SNPtype Assays and Sanger sequencing

Validation of the 125 candidate variants was performed using Fluidigm SNPtypeAssays and Sanger sequencing. Of the 125 candidates, 29 were confirmed as variants, 80 were false positive, five were validated as variants but also observed in healthy individuals, and the remaining 11 failed the validation because of low gDNA quantity from patient no. 5 (Tables 3 and 4 and S4 Table).

**Table 3:** GO and PANTHER information of genes harboring validated variants.

Gene symbol	Full gene name	PANTHER protein class	GO term	Relationship with NDs
*ABCA13*	ATP-binding cassette sub-family A member 13	ATP-binding cassette (ABC) transporter	Catalytic activity (GO:0003824) | Transporter activity (GO:0005215)	
*ACO1*	Aconitase 1, soluble	Dehydratase | Hydratase	Catalytic activity (GO:0003824)	○
*ADAM18*	ADAM metallopeptidase domain 18	–	–	
*ARAP2*	ArfGAP with RhoGAP domain, ankyrin repeat and pH domain 2	Nucleic acid binding | G-protein modulator	Binding (GO:0005488) | Catalytic activity (GO:0003824) | Enzyme regulator activity (GO:0030234)	
*ATP13A3*	ATPase type 13A3	Cation transporter | Ion channel | Hydrolase	Catalytic activity (GO:0003824) | Transporter activity (GO:0005215)	
*C1orf51 (CIART)*	Circadian-associated transcriptional repressor	–	–	
*DDX1*	DEAD (Asp-Glu-Ala-Asp) box helicase 1	RNA helicase | Helicase	Binding (GO:0005488) | Catalytic activity (GO:0003824) | Translation regulator activity (GO:0045182)	
*FAM105A*	Family with sequence similarity 105, member A	–	–	
*FGF20*	Fibroblast growth factor 20	Growth factor	Binding (GO:0005488)	○
*FNDC1*	Fibronectin type III domain containing 1	–	–	
*GBP4*	Guanylate binding protein 4	Heterotrimeric G-protein	Binding (GO:0005488) | Catalytic activity (GO:0003824)	
*GPR63*	G protein-coupled receptor 63	G-protein coupled receptor	Receptor activity (GO:0004872)	
*HACL1*	2-Hydroxyacyl-CoA lyase 1	Transferase | Dehydrogenase | Decarboxylase	Catalytic activity (GO:0003824)	
*LPA*	Lipoprotein, Lp(A)	Peptide hormone | Serine protease | Protease inhibitor | Annexin | Calmodulin	Binding (GO:0005488) | Catalytic activity (GO:0003824) | Enzyme regulator activity (GO:0030234)	○
*LRRK2*	Leucine-rich repeat kinase 2	Nonreceptor serine/threonine protein kinase | Nonreceptor tyrosine protein kinase;	Catalytic activity (GO:0003824)	○
*MYOZ2*	Myozenin 2	Structural protein	Structural molecule activity (GO:0005198)	
*PDLIM5*	PDZ and LIM domain 5	Transcription factor | Nonmotor actin binding protein	Binding (GO:0005488) | Nucleic acid binding transcription factor activity (GO:0001071) | Structural molecule activity (GO:0005198)	
*PKP1*	Plakophilin 1	Intermediate filament binding protein	Binding (GO:0005488) | Structural molecule activity (GO:0005198)	
*POLR1C*	Polymerase (RNA) I polypeptide C, 30kDa	DNA-directed RNA polymerase	Catalytic activity (GO:0003824)	
*POSTN*	Periostin, osteoblast specific factor	Signaling molecule | Cell adhesion molecule	Binding (GO:0005488)	○
*SFRP4*	Secreted Frizzled-related protein 4	Signaling molecule | G-protein coupled receptor	Binding (GO:0005488) | Receptor activity (GO:0004872)	
*SLAIN2*	SLAIN motif family, member 2	–	–	
*SLC6A5*	Solute carrier family 6 (neurotransmitter transporter), member 5	Cation transporter	SLC6A5	
*SMC5*	Structural maintenance of chromosomes 5	Nucleic acid binding	Binding (GO:0005488)	
*TET1*	Tet methylcytosine dioxygenase 1	–	–	○
*TWF1*	Twinfilin actin-binding protein 1	Nonmotor actin binding protein	Binding (GO:0005488) | Structural molecule activity (GO:0005198)	
*VEPH1*	Ventricular zone expressed pH domain-containing 1	–	–	
*WDR64*	WD repeat domain 64	–	–	
*ZRANB2*	Zinc finger, RAN-binding domain containing 2	–	–	

**Table 4:** Profiles of validated variants found only in the patients.

Chromosome	bp	rs	ref.	alt.	Amino acid change	Type	Gene	Validation methods	Observed patient no.	SIFT	PolyPhen-2
1	71542511	–	T	A	T90S	Missense	*ZRANB2*	Fluidigm	2	Tolerated, (0.12)	Benign, (0.054)
1	89657064	–	C	A	E266*	Nonsense	*GBP4*	Fluidigm, Sanger	5	-	-
1	150259289	–	A	G	K361E	Missense	*CIART*	Fluidigm	2	Tolerated, (1)	Benign, (0)
1	201285776	–	C	A	A266D	Missense	*PKP1*	Fluidigm	1	Deleterious, (0)	Possibly damaging, (0.588)
1	241946599	rs141496101	G	A	R864H	Missense	*WDR64*	Fluidigm	1	Deleterious, (0)	Probably damaging, (0.988)
2	15744615	rs137946800	G	C	V203L	Missense	*DDX1*	Fluidigm	2	Deleterious, (0.04)	Benign, (0.047)
3	15604937	rs147740656	C	G	Q544H	Missense	*HACL1*	Fluidigm	1	Deleterious, (0.03)	Possibly damaging, (0.617)
3	157160265	–	G	A	A215T	Missense	*VEPH1*	Fluidigm	4	Tolerated, (0.11)	Possibly damaging, (0.883)
3	194169200	–	A	G	I379T	Missense	*ATP13A3*	Fluidigm	1	Deleterious, (0)	Benign, (0.063)
4	36230978	–	C	T	S44N	Missense	*ARAP2*	Fluidigm	1	Tolerated, (0.15)	Benign, (0.014)
4	48422226	rs146342071	G	A	G482D	Missense	*SLAIN2*	Fluidigm	2	Deleterious, (0)	Probably damaging, (0.967)
4	95497131	–	A	G	E219G	Missense	*PDLIM5*	Fluidigm	4	Deleterious, (0)	Possibly damaging, (0.83)
4	120072108	–	G	A	R53H	Missense	*MYOZ2*	Fluidigm	2	Deleterious, (0)	Probably damaging, (1)
5	14608984	rs74815459	C	G	T252S	Missense	*FAM105A*	Fluidigm	4	Deleterious, (0.01)	Possibly damaging, (0.827)
6	43488977	–	A	AAGTACTG	K327KVL*	Nonsense	*POLR1C*	Fluidigm	2	-	-
6	97246455	–	T	C	M385V	Missense	*GPR63*	Fluidigm	4	Deleterious, (0.02)	Benign, (0.007)
6	159646580	–	C	A	R300S	Missense	*FNDC1*	Fluidigm	1	-	Probably damaging, (1)
6	161015089	–	C	T	R1177Q	Missense	*LPA*	Fluidigm	4	Deleterious, (0.03)	Probably damaging, (0.957)
7	37954042	–	T	C	I153M	Missense	*SFRP4*	Fluidigm	4	Tolerated, (0.34)	Benign, (0.001)
7	48392080	–	G	A	D3562N	Missense	*ABCA13*	Fluidigm	4	-	Probably damaging, (0.998)
8	39468116	–	A	G	H138R	Missense	*ADAM18*	Fluidigm	1	Deleterious, (0)	Probably damaging, (1)
8	16850813	rs200152641	G	C	S135C	Missense	*FGF20*	Fluidigm	2	Deleterious, (0.05)	Possibly damaging, (0.734)
9	32427337	rs189305274	G	A	V463M	Missense	*ACO1*	Fluidigm	4	Deleterious, (0.01)	Possibly damaging, (0.841)
9	72912888	–	G	T	E354*	Nonsense	*SMC5*	Fluidigm, Sanger	5	-	-
10	70333443	–	C	G	P450A	Missense	*TET1*	Fluidigm	1	Deleterious, (0.03)	Possibly damaging, (0.619)
11	20622957	–	G	A	D96N	Missense	*SLC6A5*	Fluidigm	2	Deleterious, (0.01)	Probably damaging, (1)
12	40677699	rs34410987	C	T	P755L	Missense	*LRRK2*	Fluidigm, Sanger	5	Tolerated, (0.73)	Probably damaging, (0.999)
12	44199353	–	G	GA	S38F*	Nonsense	*TWF1*	Fluidigm	1	-	-
13	38153424	rs200986202	T	A	E578V	Missense	*POSTN*	Fluidigm	1	Tolerated, (0.07)	Probably damaging, (1)

**Abbreviations:** bp, base pair; rs, reference SNP ID number; ref, reference allele; alt, alternative allele

Our analysis showed that there were 29 validated variants present in the patients in this study, with one patient harboring as many as 10 variants (patient no. 1). Each validated variant was observed in a single patient. None of the 29 variants were observed in patient no. 3. Variants with known functions with biological terms extracted from gene ontology (GO) analysis of genes harboring each variant are listed in Table 3. Four nonsense candidates were validated as variants, two of which were observed in patient no. 5, and the remaining nonsense variants were observed in patients no. 1 and 2. The other validated variants were missense variants.

### Genes harboring validated variants and analysis of biological interactions

Understanding the biological interactions among genes and diseases facilitates the identification of disease mechanisms and target proteins for new drugs. Based on such approaches, we can conveniently sort candidate genes that should be preferentially analyzed before applying other approaches using reverse genetics tools, such as methods for target gene disruption and gene silencing. Thus, genes harboring the 29 validated variants were used as seeds to analyze the biological interaction between genes and NDs, such as PrDs, AD, and PD.

The 29 genes were not found to be directly related to PrDs; however, interactions with other NDs, such as AD and PD, were detected. In addition, indirect relationships with PrDs were observed. Thus, using results from other published studies, candidate genes related to disease onset or susceptibility for CJD with the V180I mutation could be inferred.

**Fig 3:**
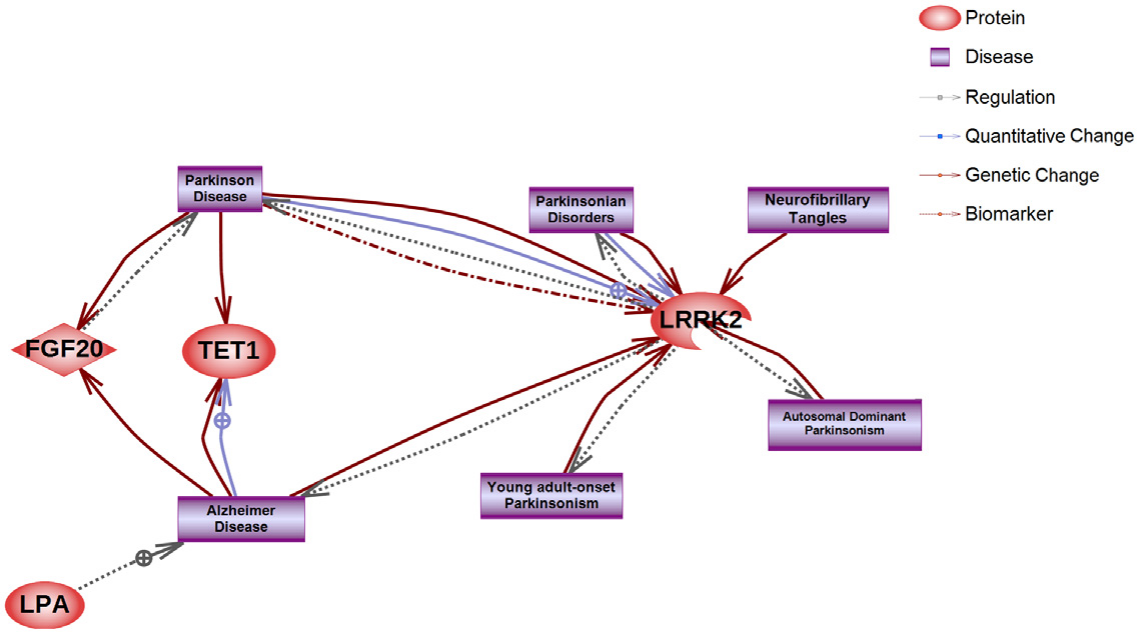
Direct interaction network of genes harboring validated variants with NDs. The red diamonds or circles indicate seed nodes (genes harboring validated variants). Each edge (biological interaction between seeds) status is explained in the legend located in the upper right panel. Terms related to AD and PD are described in purple squares. Because direct interactions of genes harboring validated variants with PrDs were not observed, the term PrDs was excluded.

**Fig 4:**
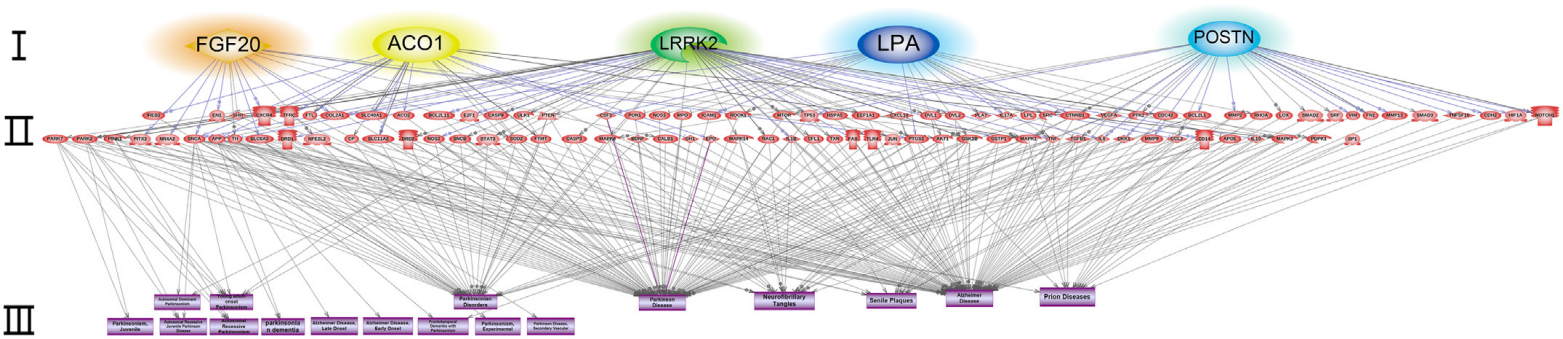
Indirect interaction network of genes harboring validated variants with NDs. Seed nodes (colored orange, yellow, green, blue, or sky blue) are described in layer I. Nodes (proteins or genes) interacting with seeds are listed in layer II. Diseases terms related to PrDs, AD, and PD are described in layer III.

Interestingly, *LPA*, *LRRK2*, *TET1*, and *FGF20* were linked directly to AD and/or PD but not to PrDs. However, genes such as *ACO1*, *POSTN*, *LRRK2*, *FGF20*, and *LPA* had indirect associations with PrDs (Figs 3 and 4, S1A–S1E Fig).

## Discussion

### Characteristics of the patients

14-3-3 protein has been used as a biomarker for CJD, and all five patients in this study were positive for 14-3-3 protein in the CSF. However, positivity for 14-3-3 protein is not always observed in gCJD patients with V180I mutation, as reported previously [37]. Alternatively, to distinguish patients with CJD from those with other diseases having elevated t-tau protein in the CSF, studies have been performed to determine the applicability of the p/t tau protein ratio [38]. In cases of CJD, the p/t tau ratio in the CSF has been shown to be significantly lower than that in patients with other diseases.

Although the diagnostic specificity (100%) of the standard RT-QuIC technique has been proven, the sensitivity of the technique in cases with the V180I mutation has been found to be somewhat lower than that in other cases of CJD [34]. In this study, the sensitivity was 75% by RT-QuIC with substrate replacement which has been used to improve the sensitivity of analysis while maintaining the high degree of specificity of standard RT-QuIC.

The diagnostic criteria for familial CJD (fCJD or gCJD) include definite or probable CJD plus definite or probable CJD in a first-degree relative and/or a neuropsychiatric disorder plus disease-specific *PRNP* gene mutation (http://www.cdc.gov/prions/cjd/diagnostic-criteria.html). Based on these diagnostic criteria, all five of the patients with gCJD in this study could be accurately diagnosed with gCJD because of their neurological symptoms and the V180I mutation, which were further supported by biochemical analyses (Tables 1 and 2).

All guardians of the five patients informed us that they did not have family histories of CJD. Although gCJD with V180I is a genetic form, it is commonly observed in families of gCJD patients with V180I without neurodegenerative symptoms. In a previous study [34], 11 of 186 gCJD patients with V180I confirmed having a family history of the disease. Three of 11 patients had family histories of CJD, and the remaining patients had family histories of dementia. Although gCJD with V180I is not frequently reported in family histories, the possibility that V180I is related to the pathogenicity of the disorder cannot be excluded because the symptoms of gCJD with the V180I mutation have been shown to be unique compared with other types of CJD and with AD [34, 36, 37]. These distinct symptoms led us to postulate that this unique pattern of symptoms and incomplete penetrance were caused by the influence of unknown genes (loci). Thus, we analyzed genomic differences between gCJD patients with V180I mutation and healthy individuals.

### Whole genome data analysis

Interestingly, in this study, each of the 29 validated variants was observed only in one patient. Therefore, we concluded that it was difficult to identify gene variants associated with the disease mechanism of CJD and found in more than two patients. Accordingly, we focused on the 29 variants observed in the single patient and analyzed the biological interactions among the gene harboring these variants by GO analysis [39]. Eleven genes were categorized in the GO term of binding (GO:0005488), and 10 were categorized in the term of catalytic activity (GO:0003824). All genes harboring nonsense mutations (*GBP4*, *POLR1C*, *SMC5*, and *TWF1*) were classified into at least one of these two GO terms. Although GO analysis provided specified categories of the core biological functions, these enrichment data could not be used to easily predict disease relationships among genes harboring validated variants.

Four newly identified nonsense variants were detected. Because these variants were observed here for the first time, no functional data for the nonsense variants were available. However, it may be possible to use reported nonsense variants in the same gene to infer the roles of the newly identified nonsense variants. As an example, SMC5, which has a role in promoting chromatid homologous recombination, harbored the variant chr9_72912888 (G to T) in patient no. 5. The *SMC5* gene encodes a protein of 1,101 amino acids, and the variant above results in the E354X mutation, encoding a stop codon. Although this variant was reported here for the first time, similar nonsense mutations in tumor samples allow us to infer the phenotype [40]. Additionally, GBP4, an interferon signaling-related protein, harbored the newly identified variant chr1_89657064 (C to A) in the same patient, resulting in the E266X mutation. Sixteen stop gain mutations have been reported, as listed in the Ensembl database (GRCh37, release 82). These mutations have been observed in cancer tissues of uterine corpus endonetroioid carcinoma and lung squamous cell carcinoma. *POLR1C*, a subunit of both RNA polymerase I and III, contained a newly identified nonsense variant (chr6_43488977, which creates K327KVL*) in patient no. 2. Seven similar nonsense mutations, detected in cancer tissues, are listed in the Ensembl database (GRCh37, release 82), and dysfunctions in POLR1C activity are associated with Treacher Collins syndrome. Finally, *TWF1*, an actin monomer binding protein, harbored the newly identified variant chr12_44199353 (S38F*) in patient no. 1. Five similar nonsense variants in *TWF1* are listed in the Ensembl database (GRCh37, release 82) and were observed in tumors of the large intestine and lung.

Changes in other nucleotides causing nonsense mutations in these four genes have not been reported to have any significant relationships with NDs. The four newly identified variants causing nonsense mutations must be analyzed further to determine their relationships with prion diseases and other NDs using reverse genetic tools, such as gene knockout or gene interference technology.

### Genes harboring validated variants and analysis of biological interactions

LPA has been shown to be associated with AD (Figs 3 and 4 and S1A Fig), and we observed a newly identified *LPA* variant (chr6_161015089, R1177Q) in patient no. 4 [41, 42]. Although this mutation is not a form related to the nonsense phenotype, PolyPhen-2 and Sorting Intolerant From Tolerant (SIFT) scores suggested that the mutation caused an abnormal functional change in LPA in patient no. 4 (Table 4).

The single-nucleotide polymorphism (SNP) rs189305274, which was observed in patient no. 4, is a variant in *ACO1*. The abnormal functional change in this target resulting from rs189305274 was predicted to be damaging by PolyPhen-2 and SIFT scores (Table 4). ACO1 was not shown to be directly associated with NDs. However, indirect interactions with NDs, including PrDs, were detected. ACO1 has been reported to inhibit or regulate amyloid beta (A4) precursor protein (APP) [43, 44], which is involved in the induction of AD and acceleration of PrDs [27, 45, 46]. Thus, mutations that cause functional changes in ACO1 may delay PrDs progression by regulating APP. These results may explain why patient no. 4 is still alive and suggest that factors inhibiting ACO1 expression may be promising candidates for repression of CJD.

The S135C mutation (rs200152641) in *FGF20* was observed in patient no. 2. FGF20, which was found to interact directly with PD, has been shown to be a candidate therapeutic protein for PD (Fig 3). An indirect interaction was observed between FGF20 and PrDs (Fig 4 and S1C Fig), and mitogen-activated protein kinase (MAPK) 1 and MAPK3 have been reported to inhibit PrP expression, supporting their potential application in therapeutic strategies against PrDs. Because FGF20 is known to induce MAPK1 and MAPK3, it may also have applications in the treatment of PrDs. Significant functional changes in FGF20 protein were predicted by PolyPhen-2 and SIFT (Table 4); such changes may influence MAPK1 and MAPK3 regulation, representing a potential therapeutic strategy for the management of PrDs. Thus, this variant may has influenced the rapid progression of patient no. 2. POSTN and LRRK2 are also involved in the upregulation and activation of MAPK1 and/or MAPK3, respectively (S1B and S1E Fig).

TET1 is associated with late-onset AD (LOAD), and the expression of TET1 is increased in the hippocampus of patients with preclinical AD and LOAD [47], and the exon region of *TET1* (also called CXXC finger 6 [*CXXC6*]) has been shown to be related to LOAD [48]. However, although TET1 has been shown to be directly related to AD, we did not detect a direct or indirect relationship with PrDs.

The E578V mutation in *POSTN* was observed in patient no. 1. POSTN has been reported to promote upregulation of NOTCH-1 [49, 50]. Moreover, NOTCH-1 activation is associated with dendritic atrophy in PrDs, and NOTCH-1 regulation may be controlled by prion infection [51].

LRRK2 has been reported to be related to AD. The SNP rs34410987, which causes a proline-to-leucine mutation (P755L) in *LRRK2*, as observed in this study, was first reported in nine Chinese patients with PD in 2006 and is an important mutation in the diagnosis of PD [52]. However, it is unclear whether this mutation is a causative agent for PD because it has also been observed in healthy individuals, and no significant associations with PD has been reported [53–56]. Despite these previous findings, we could not exclude the possibility that P755L in LRRK2 may be associated with PrDs. Further studies are needed to analyze the associations between diseases and gene variations. For example, in gastric cancer [57], genotype associations have been analyzed among various types of gastric cancer according to the Laurens’ classification system. Additionally, to determine whether P755L may be a causative mutation in PrDs, further analyses are needed. The occurrence of both V180I in *PRNP* and P755L in *LRRK2* may represent a causative haplotype for gCJD. However, the number of patient samples was insufficient to confirm this possibility, and additional studies will be required.

## Conclusions

Our results supported the possibility that PrDs may be associated with other NDs through the different gene variants found in five gCJD patients with V180I mutation, including a patient who survived more than 10 years after disease diagnosis. Six of the validated variants were located within genes (*LPA*, *LRRK2*, *TET1*, *FGF20*, *ACO1*, and *POSTN*) that have been shown to be associated with NDs such as PrDs, AD, and PD. Although these gene mutations having significant associations with NDs were observed only in patients with gCJD, more in-depth follow-up studies of the families of these patients are required to definitively conclude that these variants are involved in the pathogenesis of gCJD with V180I. Four variants causing nonsense mutations were newly identified. Further studies are needed to determine the relationships between these mutations and disease development because of the lack of significant phenotype data on the genes harboring these variants (e.g., *SMC5*, *GBP4*, *POLR1C*, and *TWF1*). Despite the lack of data, we cannot exclude the possibility that these genes may contribute to the overall risk of the disease. In order to confirm these data, additional analyses using reverse genetic methods, such as gene knockout or gene interference technologies, are needed.

## Materials and Methods

This study was approved by the Institutional Review Board of the Korea Centers for Disease Control and Prevention (IRB No. 2014-06EXP-04-R-A). Written informed consent was obtained from the patients or their legal guardians.

### Subjects

All five gCJD patients with V180I mutation described in this study were South Korean natives. Detail information regarding these patients is given in Tables 1 and 2. No patients reported having a family history of CJD. In addition, we also selected the whole genome sequences of 135 healthy Koreans stored at the Division of Center for Genome Science in KNIH as controls for variation filtering. However, the mutation V180I was observed in one individual. Although this individual did not have gCJD, the genomic sequence of this individual was excluded. Because DNA samples of these controls were not available for further analysis, we also collected DNA samples from 10 healthy individual volunteers (ages 20–40 years) for variant validation. Thus, a total of 145 DNA samples from healthy Koreans were used in this study.

### DNA extraction

Genomic DNA was extracted from the patients and 10 control individuals described above. Total DNA was isolated from whole blood samples using a QIAamp DNA Blood Mini Kit (Qiagen, Hilden, Germany), according to the manufacturer’s instructions. The extracted DNA was quantified using a Quant-iT PicoGreen dsDNA kit (Invitrogen, Carlsbad, CA, USA).

### Biochemical analysis of the CSF of patients with gCJD

#### Detection of 14-3-3 and tau protein

CSF samples from all five patients were analyzed by western blot analysis for 14-3-3 protein expression. Enzyme-linked immunosorbent assays (ELISA) were used to analyze t-tau and p-tau proteins. These biochemical analyses were performed as previously described [58].

#### Detection of PrP^Sc^ using RT-QuIC with substrate replacement

Purification of recombinant human PrP (rHuPrP: residues 23–231, codon 129M) was performed as previously described [59]. After purification, rHuPrP was stored at -80°C. Brain homogenates (10% [w/v]) were diluted (10^−3^) with 0.1% SDS/PBS/N2. Next, 85 μL of RT-QuIC buffer mixture (300 mM NaCl, 160 mM phosphate buffer, 10 μM thioflavin T (ThT), 1 mM EDTA, and 1 mg rHuPrP) was loaded into each well of a 96-well Optical Bottom Plate (Nalge Nunc International) and mixed with 15 μL of a CSF sample with 1:10 dilution or diluted brain homogenate. The assay was monitored using an OPTIMA FLUOstar plate reader at 42°C for over 118 h. ThT fluorescence measurements (440 nm excitation and 485 nm emission) were acquired every 42 min. RT-QuIC buffer was replaced with fresh RT-QuIC buffer mixture (as described above) after 48 h. Two human brain homogenates (NHNX0/0001 and NHBZ0/0005) were obtained from National Institute for Biological Standards and Control.

#### Whole genome sequencing

Whole genome sequencing of the five gCJD patients with V180I (cases 1–5) was performed using the Solexa sequencing technology platform (HiSeq2500; Illumina, San Diego, CA, USA). Briefly, 1–3 μg of gDNA was fragmented by sonication. Library span size was controlled using a Covaris System to generate ~500-bp inserts. The sonicated DNA was end-repaired using T4 DNA polymerase and Klenow polymerase. Tailing was performed to create sticky ends, and Illumina paired-end adaptor oligonucleotides were ligated to the sticky ends. Adaptor-ligated oligonucleotides were subjected to polymerase chain reaction (PCR) enrichment. Ligated DNA was then size-selected for lengths of about 500 bp, including adaptors of about 180 bp. Cluster generation was performed using amplified library DNA in a flowcell to produce clusters. Purified fragments were loaded onto the Illumina flow cell for sequencing on the Illumina Hiseq2500 instrument.

Raw data are described in S1 and S2 Tables. Reads were aligned to the Human Reference Genome (hg19) using Burrows-Wheeler Aligner (BWA) v0.6.1. Multisample SNP calling was performed using GATK and Picard tools with default parameters.

The genome sequences of the 134 healthy individuals were called and annotated in a manner similar to that of the patients. Next, variant call format (VCF) files were merged using VCF tools v4.1 (https://vcftools.githubio.io/). Differences in variant counts between groups were calculated and analyzed using PLINK v1.9 (pngu.mgh.harvard.edu/~purcell/plink).

#### Variant filtering

A total of 11,223,176 candidate variants without rs number were identified, of which 802,183 variants were not observed in 134 healthy Korean sequences. Next, intronic variants were removed, and variants causing frameshift, inframe-deletion, missense, or stop loss mutations were selected. Additionally, 7,425,674 candidate variants with rs numbers were filtered in the same manner. Next, unannotated variants in any gene and intronic variants were removed, and 1,102 candidate exonic variants, including variants causing frameshift, inframe-deletion, missense, or stop loss mutations, were selected for further validation. Of these 1,102 candidates, 110 were found in all five of the patients, and 94 newly identified variants were also selected. Additionally, 20 variants found in 1–4 patients were selected followed by phred scores (over 600). These 224 candidates were selected for variant validation; and after filtering, as described in S4 Table, 125 candidates were processed for further validation.

#### Validation of selected variants

A total of 125 variants were validated by Fluidigm BioMark Dynamic Array. Variant validation was performed among all five patients and 10 healthy individuals as described above. However, variant validation in patient no. 5 failed. Thus, the variants observed in patient no. 5 were revalidated by Sanger sequencing. However, owing to low gDNA quality, only three variants were revalidated using this method. PCR and sequencing were performed as previously described using the PCR primers listed in S5 Table [23].

#### Genome data analysis

All variants that showed different allele frequencies between groups were analyzed using Variant Effect Predictor in ENSEMBL release 81 (based on the GRCh37), and the effects of variants on the annotated canonical transcripts for all genes were assessed using PolyPhen-2 and SIFT [60, 61]. The effects of amino acids substitutions resulting from the different variants were predicted and considered as damaging changes if indicated as ‘probably damaging’ or ‘possibly damaging’ by PolyPhen-2 or if indicated as ‘deleterious’ by SIFT. GO categorization were performed using the Panther Classification System (http://pantherdb.org; Table 3). Known information of analyzed genes was extracted from GeneCards (http://www.genecards.org).

#### Biological network analysis

Biological networks among the genes (proteins) harboring validated variants were created using Pathway Studio 9.0 software (Ariadne, Rockville, MD, USA). The molecular interactions between the genes and NDs, such as AD, PD, and PrDs, were extracted from Elsevier's MedScan text-mining software, which contains biological articles and abstracts. Error information and interactions because of mismatches and mistyping were removed from the output data.

## Supporting Information

S1 Fig Indirect interaction of genes with PrDs, AD, and PD. (A) Indirect interactions of *LPA*, (B) *LRRK2*, (C) *FGF20*, (D) *ACO1*, and (E) *POSTN*.
(TIF)

S1 File Genomic information for the healthy individual with V180I. (DOCX)

S1 Table Raw data for whole genome sequences. (DOCX)

S2 Table Alignment summary of raw genome data. (DOCX)

S3 Table Information of variants in *PRNP* observed in one healthy individual with V180I. (XLSX)

S4 Table Information for 1,102 variants. All information for the 1,102 variants, including variants generated during the validation process (see stage IV and V in Fig 3). The Fluidigm array design results are listed in the last two columns. Ninety-nine variants were not designed for Fluidigm SNPtypeAssays for the following reasons: the target design parameters were not met, and there were regions containing large repeats and/or complex genomic diversity; GC contents were outside of the range of product specification; adjacent variant(s) were found within 30 bp of the target SNP; and target sequences were not supported. ‘High’ indicates that the target could be designed to the product specifications; ‘Medium’ indicates that the target could be designed, but with limited support because of problems such as high GC content (over 65%) of the target or indel target.
(XLSX)

S5 Table Primer sequences for variants validation. (DOCX)

## References

[1] Prusiner SB. Novel proteinaceous infectious particles cause scrapie. Science. 1982;216(4542):136–44. 6801762 10.1126/science.6801762

[2] Kitamoto T, Muramoto T, Mohri S, Doh-Ura K, Tateishi J. Abnormal isoform of prion protein accumulates in follicular dendritic cells in mice with Creutzfeldt-Jakob disease. J Virol. 1991;65(11):6292–5. 1681118 10.1128/jvi.65.11.6292-6295.1991 PMC250334

[3] Collins S, McLean CA, Masters CL. Gerstmann-Straussler-Scheinker syndrome,fatal familial insomnia, and kuru: a review of these less common human transmissible spongiform encephalopathies. J Clin Neurosci. 2001;8(5):387–97. 11535002 10.1054/jocn.2001.0919

[4] Doh-ura K, Tateishi J, Sasaki H, Kitamoto T, Sakaki Y. Pro—leu change at position 102 of prion protein is the most common but not the sole mutation related to Gerstmann-Straussler syndrome. Biochem Biophys Res Commun. 1989;163(2):974–9. 2783132 10.1016/0006-291x(89)92317-6

[5] Kitamoto T, Amano N, Terao Y, Nakazato Y, Isshiki T, Mizutani T, et al A new inherited prion disease (PrP-P105L mutation) showing spastic paraparesis. Ann Neurol. 1993;34(6):808–13. 8250529 10.1002/ana.410340609

[6] Goldfarb LG, Brown P, Haltia M, Cathala F, McCombie WR, Kovanen J, et al Creutzfeldt-Jakob disease cosegregates with the codon 178Asn PRNP mutation in families of European origin. Ann Neurol. 1992;31(3):274–81. 1353341 10.1002/ana.410310308

[7] Goldfarb LG, Haltia M, Brown P, Nieto A, Kovanen J, McCombie WR, et al New mutation in scrapie amyloid precursor gene (at codon 178) in Finnish Creutzfeldt-Jakob kindred. Lancet. 1991;337(8738):425. 10.1016/0140-6736(91)91198-4 1671440

[8] Kitamoto T, Ohta M, Doh-ura K, Hitoshi S, Terao Y, Tateishi J. Novel missense variants of prion protein in Creutzfeldt-Jakob disease or Gerstmann-Straussler syndrome. Biochem Biophys Res Commun. 1993;191(2):709–14. 8461023 10.1006/bbrc.1993.1275

[9] Goldgaber D, Goldfarb LG, Brown P, Asher DM, Brown WT, Lin S, et al Mutations in familial Creutzfeldt-Jakob disease and Gerstmann-Straussler-Scheinker's syndrome. Exp Neurol. 1989;106(2):204–6. 2572450 10.1016/0014-4886(89)90095-2

[10] Peoc'h K, Manivet P, Beaudry P, Attane F, Besson G, Hannequin D, et al Identification of three novel mutations (E196K, V203I, E211Q) in the prion protein gene (PRNP) in inherited prion diseases with Creutzfeldt-Jakob disease phenotype. Hum Mutat. 2000;15(5):482. 10.1002/(SICI)1098-1004(200005)15:5<482::AID-HUMU16>3.0.CO;2-1 10790216

[11] Kobayashi S, Saito Y, Maki T, Murayama S. Cortical propagation of Creutzfeldt-Jakob disease with codon 180 mutation. Clin Neurol Neurosurg. 2010;112(6):520–3. 10.1016/j.clineuro.2010.03.015 20409635

[12] Mead S. Prion disease genetics. Eur J Hum Genet. 2006;14(3):273–81. 16391566 10.1038/sj.ejhg.5201544

[13] Mastrianni JA. The genetics of prion diseases. Genet Med. 2010;12(4):187–95. 10.1097/GIM.0b013e3181cd7374 20216075

[14] Tateishi J, Kitamoto T. Inherited prion diseases and transmission to rodents. Brain Pathol. 1995;5(1):53–9. 7767491 10.1111/j.1750-3639.1995.tb00577.x

[15] Tateishi J, Kitamoto T, Hoque MZ, Furukawa H. Experimental transmission of Creutzfeldt-Jakob disease and related diseases to rodents. Neurology. 1996;46(2):532–7. 8614527 10.1212/wnl.46.2.532

[16] Dickinson AG, Mackay JM. Genetical Control of the Incubation Period in Mice of the Neurological Disease, Scrapie. Heredity (Edinb). 1964;19:279–88. 14167576 10.1038/hdy.1964.31

[17] Carlson GA, Kingsbury DT, Goodman PA, Coleman S, Marshall ST, DeArmond S, et al Linkage of prion protein and scrapie incubation time genes. Cell. 1986;46(4):503–11. 3015416 10.1016/0092-8674(86)90875-5

[18] Westaway D, Goodman PA, Mirenda CA, McKinley MP, Carlson GA, Prusiner SB. Distinct prion proteins in short and long scrapie incubation period mice. Cell. 1987;51(4):651–62. 2890436 10.1016/0092-8674(87)90134-6

[19] Kingsbury DT, Kasper KC, Stites DP, Watson JD, Hogan RN, Prusiner SB. Genetic control of scrapie and Creutzfeldt-Jakob disease in mice. J Immunol. 1983;131(1):491–6. 6408182

[20] Lloyd SE, Onwuazor ON, Beck JA, Mallinson G, Farrall M, Targonski P, et al Identification of multiple quantitative trait loci linked to prion disease incubation period in mice. Proc Natl Acad Sci U S A. 2001;98(11):6279–83. 11353827 10.1073/pnas.101130398 PMC33459

[21] Lloyd SE, Uphill JB, Targonski PV, Fisher EM, Collinge J. Identification of genetic loci affecting mouse-adapted bovine spongiform encephalopathy incubation time in mice. Neurogenetics. 2002;4(2):77–81. 12481985 10.1007/s10048-002-0133-9

[22] Hsiao K, Meiner Z, Kahana E, Cass C, Kahana I, Avrahami D, et al Mutation of the prion protein in Libyan Jews with Creutzfeldt-Jakob disease. N Engl J Med. 1991;324(16):1091–7. 2008182 10.1056/NEJM199104183241604

[23] Lee SM, Chung M, Hwang KJ, Ju YR, Hyeon JW, Park JS, et al Biological network inferences for a protection mechanism against familial Creutzfeldt-Jakob disease with E200K pathogenic mutation. BMC Med Genomics. 2014;7:52 10.1186/1755-8794-7-52 25149502 PMC4151374

[24] Hainfellner JA, Wanschitz J, Jellinger K, Liberski PP, Gullotta F, Budka H. Coexistence of Alzheimer-type neuropathology in Creutzfeldt-Jakob disease. Acta Neuropathol. 1998;96(2):116–22. 9705125 10.1007/s004010050870

[25] Woerman AL, Stohr J, Aoyagi A, Rampersaud R, Krejciova Z, Watts JC, et al Propagation of prions causing synucleinopathies in cultured cells. Proc Natl Acad Sci U S A. 2015;112(35):E4949–58. 10.1073/pnas.1513426112 26286986 PMC4568231

[26] Prusiner SB, Woerman AL, Mordes DA, Watts JC, Rampersaud R, Berry DB, et al Evidence for alpha-synuclein prions causing multiple system atrophy in humans with parkinsonism. Proc Natl Acad Sci U S A. 2015;112(38):E5308–17. 10.1073/pnas.1514475112 26324905 PMC4586853

[27] Morales R, Estrada LD, Diaz-Espinoza R, Morales-Scheihing D, Jara MC, Castilla J, et al Molecular cross talk between misfolded proteins in animal models of Alzheimer's and prion diseases. J Neurosci. 2010;30(13):4528–35. 10.1523/JNEUROSCI.5924-09.2010 20357103 PMC2859074

[28] Jucker M, Walker LC. Self-propagation of pathogenic protein aggregates in neurodegenerative diseases. Nature. 2013;501(7465):45–51. 10.1038/nature12481 24005412 PMC3963807

[29] Grundke-Iqbal I, Iqbal K, Quinlan M, Tung YC, Zaidi MS, Wisniewski HM. Microtubule-associated protein tau. A component of Alzheimer paired helical filaments. J Biol Chem. 1986;261(13):6084–9. 3084478

[30] Narhi L, Wood SJ, Steavenson S, Jiang Y, Wu GM, Anafi D, et al Both familial Parkinson's disease mutations accelerate alpha-synuclein aggregation. J Biol Chem. 1999;274(14):9843–6. 10092675 10.1074/jbc.274.14.9843

[31] Adjou KT, Allix S, Ouidja MO, Backer S, Couquet C, Cornuejols MJ, et al Alpha-synuclein accumulates in the brain of scrapie-affected sheep and goats. J Comp Pathol. 2007;137(1):78–81. 17544436 10.1016/j.jcpa.2007.03.007

[32] Chasseigneaux S, Haik S, Laffont-Proust I, De Marco O, Lenne M, Brandel JP, et al V180I mutation of the prion protein gene associated with atypical PrPSc glycosylation. Neurosci Lett. 2006;408(3):165–9. 17029785 10.1016/j.neulet.2006.08.008

[33] Kovacs GG, Puopolo M, Ladogana A, Pocchiari M, Budka H, van Duijn C, et al Genetic prion disease: the EUROCJD experience. Hum Genet. 2005;118(2):166–74. 16187142 10.1007/s00439-005-0020-1

[34] Qina T, Sanjo N, Hizume M, Higuma M, Tomita M, Atarashi R, et al Clinical features of genetic Creutzfeldt-Jakob disease with V180I mutation in the prion protein gene. BMJ Open. 2014;4(5):e004968 10.1136/bmjopen-2014-004968 24838726 PMC4025468

[35] Lee SM, Ju YR, Choi BY, Hyeon JW, Park JS, Kim CK, et al Genotype patterns and characteristics of PRNP in the Korean population. Prion. 2012;6(4):375–82. 10.4161/pri.20195 22561193 PMC3609067

[36] Jin K, Shiga Y, Shibuya S, Chida K, Sato Y, Konno H, et al Clinical features of Creutzfeldt-Jakob disease with V180I mutation. Neurology. 2004;62(3):502–5. 14872044 10.1212/01.wnl.0000106954.54011.80

[37] Mutsukura K, Satoh K, Shirabe S, Tomita I, Fukutome T, Morikawa M, et al Familial Creutzfeldt-Jakob disease with a V180I mutation: comparative analysis with pathological findings and diffusion-weighted images. Dement Geriatr Cogn Disord. 2009;28(6):550–7. 10.1159/000254842 20051687 PMC2837892

[38] Satoh K, Shirabe S, Eguchi H, Tsujino A, Eguchi K, Satoh A, et al 14-3-3 protein, total tau and phosphorylated tau in cerebrospinal fluid of patients with Creutzfeldt-Jakob disease and neurodegenerative disease in Japan. Cell Mol Neurobiol. 2006;26(1):45–52. 16633900 10.1007/s10571-006-9370-z PMC11521384

[39] Ashburner M, Ball CA, Blake JA, Botstein D, Butler H, Cherry JM, et al Gene ontology: tool for the unification of biology. The Gene Ontology Consortium. Nat Genet. 2000;25(1):25–9. 10802651 10.1038/75556 PMC3037419

[40] Guo G, Sun X, Chen C, Wu S, Huang P, Li Z, et al Whole-genome and whole-exome sequencing of bladder cancer identifies frequent alterations in genes involved in sister chromatid cohesion and segregation. Nat Genet. 2013;45(12):1459–63. 10.1038/ng.2798 24121792 PMC7512009

[41] Compton D, Wavrant DeVrieze F, Petersen RC, Tangalos E, Li L, Hardy J. Possible association between genetic variability at the apolipoprotein(a) locus and Alzheimer's disease in apolipoprotein E2 carriers. Neurosci Lett. 2002;331(1):60–2. 12359323 10.1016/s0304-3940(02)00703-6

[42] Solfrizzi V, Panza F, D'Introno A, Colacicco AM, Capurso C, Basile AM, et al Lipoprotein(a), apolipoprotein E genotype, and risk of Alzheimer's disease. J Neurol Neurosurg Psychiatry. 2002;72(6):732–6. 12023414 10.1136/jnnp.72.6.732 PMC1737901

[43] Cirillo D, Agostini F, Klus P, Marchese D, Rodriguez S, Bolognesi B, et al Neurodegenerative diseases: quantitative predictions of protein-RNA interactions. RNA. 2013;19(2):129–40. 10.1261/rna.034777.112 23264567 PMC3543085

[44] Cahill CM, Lahiri DK, Huang X, Rogers JT. Amyloid precursor protein and alpha synuclein translation, implications for iron and inflammation in neurodegenerative diseases. Biochim Biophys Acta. 2009;1790(7):615–28. 10.1016/j.bbagen.2008.12.001 19166904 PMC3981543

[45] Yu L, Wang S, Chen X, Yang H, Li X, Xu Y, et al Orientin alleviates cognitive deficits and oxidative stress in Abeta1-42-induced mouse model of Alzheimer's disease. Life Sci. 2015;121:104–9. 10.1016/j.lfs.2014.11.021 25497709

[46] Tai LM, Bilousova T, Jungbauer L, Roeske SK, Youmans KL, Yu C, et al Levels of soluble apolipoprotein E/amyloid-beta (Abeta) complex are reduced and oligomeric Abeta increased with APOE4 and Alzheimer disease in a transgenic mouse model and human samples. J Biol Chem. 2013;288(8):5914–26. 10.1074/jbc.M112.442103 23293020 PMC3581407

[47] Bradley-Whitman MA, Lovell MA. Epigenetic changes in the progression of Alzheimer's disease. Mech Ageing Dev. 2013;134(10):486–95. 10.1016/j.mad.2013.08.005 24012631 PMC3857018

[48] Morgan AR, Hamilton G, Turic D, Jehu L, Harold D, Abraham R, et al Association analysis of 528 intra-genic SNPs in a region of chromosome 10 linked to late onset Alzheimer's disease. Am J Med Genet B Neuropsychiatr Genet. 2008;147B(6):727–31. 10.1002/ajmg.b.30670 18163421

[49] Merle B, Garnero P. The multiple facets of periostin in bone metabolism. Osteoporos Int. 2012;23(4):1199–212. 10.1007/s00198-011-1892-7 22310955

[50] Wang MM. Notch signaling and Notch signaling modifiers. Int J Biochem Cell Biol. 2011;43(11):1550–62. 10.1016/j.biocel.2011.08.005 21854867 PMC3395424

[51] Ishikura N, Clever JL, Bouzamondo-Bernstein E, Samayoa E, Prusiner SB, Huang EJ, et al Notch-1 activation and dendritic atrophy in prion disease. Proc Natl Acad Sci U S A. 2005;102(3):886–91. 15640354 10.1073/pnas.0408612101 PMC545568

[52] Wu T, Zeng Y, Ding X, Li X, Li W, Dong H, et al A novel P755L mutation in LRRK2 gene associated with Parkinson's disease. Neuroreport. 2006;17(18):1859–62. 17179858 10.1097/WNR.0b013e328010521c

[53] Tan EK, Lim HQ, Yuen Y, Zhao Y. Pathogenicity of LRRK2 P755L variant in Parkinson's disease. Mov Disord. 2008;23(5):734–6. 10.1002/mds.21852 18265005

[54] Di Fonzo A, Wu-Chou YH, Lu CS, van Doeselaar M, Simons EJ, Rohe CF, et al A common missense variant in the LRRK2 gene, Gly2385Arg, associated with Parkinson's disease risk in Taiwan. Neurogenetics. 2006;7(3):133–8. 16633828 10.1007/s10048-006-0041-5

[55] Tomiyama H, Mizuta I, Li Y, Funayama M, Yoshino H, Li L, et al LRRK2 P755L variant in sporadic Parkinson's disease. J Hum Genet. 2008;53(11–12):1012–5. 10.1007/s10038-008-0336-5 18923807

[56] Wu X, Tang KF, Li Y, Xiong YY, Shen L, Wei ZY, et al Quantitative assessment of the effect of LRRK2 exonic variants on the risk of Parkinson's disease: a meta-analysis. Parkinsonism Relat Disord. 2012;18(6):722–30. 10.1016/j.parkreldis.2012.04.013 22575234

[57] Wang K, Yuen ST, Xu J, Lee SP, Yan HH, Shi ST, et al Whole-genome sequencing and comprehensive molecular profiling identify new driver mutations in gastric cancer. Nat Genet. 2014;46(6):573–82. 10.1038/ng.2983 24816253

[58] Hyeon JW, Kim SY, Lee J, Park JS, Hwang KJ, Lee SM, et al Alternative application of Tau protein in Creutzfeldt-Jakob disease diagnosis: Improvement for weakly positive 14-3-3 protein in the laboratory. Sci Rep. 2015;5:15283 10.1038/srep15283 26507666 PMC4623667

[59] Atarashi R, Moore RA, Sim VL, Hughson AG, Dorward DW, Onwubiko HA, et al Ultrasensitive detection of scrapie prion protein using seeded conversion of recombinant prion protein. Nat Methods. 2007;4(8):645–50. 17643109 10.1038/nmeth1066

[60] Adzhubei I, Jordan DM, Sunyaev SR. Predicting functional effect of human missense mutations using PolyPhen-2. Curr Protoc Hum Genet. 2013;Chapter 7:Unit7 20 10.1002/0471142905.hg0720s76 23315928 PMC4480630

[61] Kumar P, Henikoff S, Ng PC. Predicting the effects of coding non-synonymous variants on protein function using the SIFT algorithm. Nat Protoc. 2009;4(7):1073–81. 10.1038/nprot.2009.86 19561590

