# Supplementary figures and images for: Genomic Characteristics of Genetic Creutzfeldt-Jakob Disease Patients with V180I Mutation and Associations with Other Neurodegenerative Disorders
Source: PLoS One. 2016 Jun 24;11(6):e0157540. doi: 10.1371/journal.pone.0157540 (PMC4920420; doi:10.1371/journal.pone.0157540)

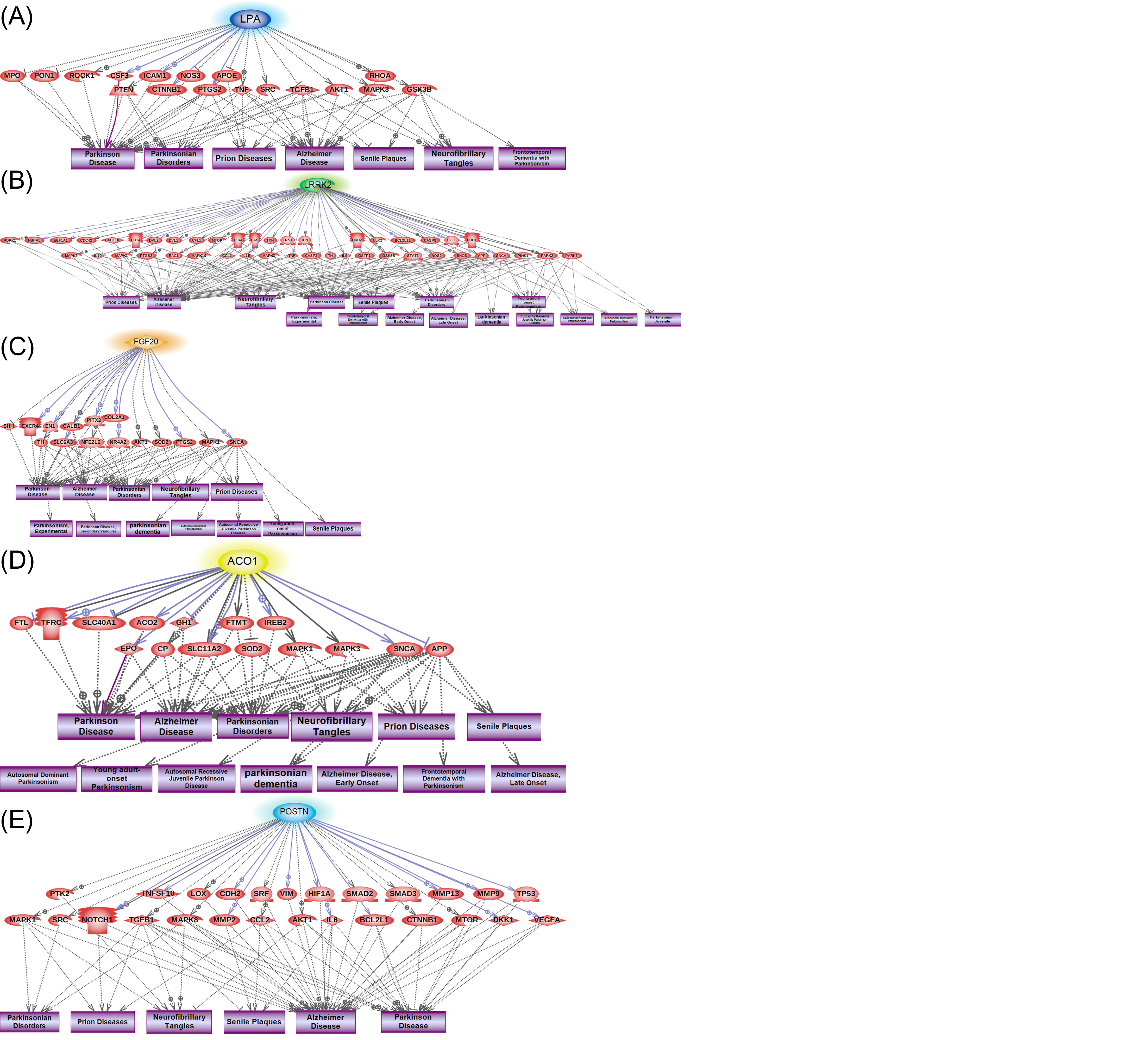

Supplement: S1 Fig — (A) Indirect interactions of LPA, (B) LRRK2, (C) FGF20, (D) ACO1, and (E) POSTN. (TIF) [file pone.0157540.s001.tif]
