# Supplementary material for: Genomic Characteristics of Genetic Creutzfeldt-Jakob Disease Patients with V180I Mutation and Associations with Other Neurodegenerative Disorders
Source: PLoS One. 2016 Jun 24;11(6):e0157540. doi: 10.1371/journal.pone.0157540 (PMC4920420; doi:10.1371/journal.pone.0157540)
Supplement: S1 File — (DOCX) [file pone.0157540.s002.docx]

**S1 File.** Genomic information for the healthy individual with V180I*.*

One of the 135 healthy individuals carried a V180I mutation; thus, the genomic data for this individual were excluded in the comparison of variants between gCJD patients and healthy individuals. The genomic information for this individual is included in S3 Table. None of the 29 variants listed in Table 4 were observed in one healthy individual carrying the V180I mutation in *PRNP*. Interestingly, five variants having amino acid changes of G40G, P68P, M129V, V180I, and E219K in the protein-coding region were observed. Additionally, 35 intron variants were observed. In these variants, rs57633656 has been shown to have strong linkage disequilibrium (D′ = 0.94, *r*^2^ = 0.89) with rs1800014 located in the 219 codon-coding region, which led to an amino acid change (GAG to AAG, E to K), even though the physical distance between the two sites was 14,142 bp [1].

Twenty-two missense variants were observed in genes related to type 1 diabetes (*HLA-DQA1*, *HLA-DRB1*, *HLA-C*, *LTA*, and *HLA-DPA1*). Additionally, one missense variant was observed in Huntington-associated protein 1 (HAP1).

Although scientific interpretations of the functions of the variants and genes described above are limited because variant validations were not performed and this individual did not show the pathological phenotype for CJD, it is interesting that M129V, V180I, and E219K were detected concurrently in one individual.

Reference

1. Lee SM, Ju YR, Choi BY, Hyeon JW, Park JS, Kim CK, et al. Genotype patterns and characteristics of PRNP in the Korean population. Prion. 2012;6(4):375-82.
