# Supplementary material for: Genomic Characteristics of Genetic Creutzfeldt-Jakob Disease Patients with V180I Mutation and Associations with Other Neurodegenerative Disorders
Source: PLoS One. 2016 Jun 24;11(6):e0157540. doi: 10.1371/journal.pone.0157540 (PMC4920420; doi:10.1371/journal.pone.0157540)
Supplement: S1 Table — (DOCX) [file pone.0157540.s003.docx]

**S1 Table. Raw data for whole genome sequences**

| **Patient**  **No.** | **read count** | **read length** | **reads include 'N'** | **reads include 'N'** | **total N** | **total N** | **Q30** | **Q20** | **GC** |
| --- | --- | --- | --- | --- | --- | --- | --- | --- | --- |
| 1 | 993,376,206 | 125,165,401,956 | 2,030,003 | 0.0016% | 48,716,605 | 0.0389% | 113,652,403,669 | 118,033,609,866 | 39.39% |
| 2 | 816,572,016 | 102,888,074,016 | 1,640,280 | 0.0016% | 39,401,643 | 0.0383% | 92,950,765,764 | 96,702,279,684 | 39.55% |
| 3 | 624,374,450 | 78,671,180,700 | 1,280,565 | 0.0016% | 30,715,693 | 0.0390% | 71,531,451,371 | 74,238,937,524 | 39.42% |
| 4 | 867,041,654 | 109,247,248,404 | 1,772,656 | 0.0016% | 42,465,960 | 0.0389% | 98,651,543,741 | 102,549,646,731 | 39.65% |
| 5 | 817,944,906 | 103,061,058,156 | 1,664,526 | 0.0016% | 40,053,905 | 0.0389% | 93,431,045,424 | 97,056,074,565 | 39.52% |
