# Supplementary material for: Genomic Characteristics of Genetic Creutzfeldt-Jakob Disease Patients with V180I Mutation and Associations with Other Neurodegenerative Disorders
Source: PLoS One. 2016 Jun 24;11(6):e0157540. doi: 10.1371/journal.pone.0157540 (PMC4920420; doi:10.1371/journal.pone.0157540)
Supplement: S2 Table — (DOCX) [file pone.0157540.s004.docx]

**S2 Table. Alignment summary of raw genome data**

|  | **Patient no.** | | | | |
| --- | --- | --- | --- | --- | --- |
|  | **1** | **2** | **3** | **4** | **5** |
| PF (passing Illumina`s filter) reads aligned | 796,617,350 | 677,915,830 | 444,642,218 | 701,746,916 | 703,829,828 |
| % of PF reads aligned | 98.93 % | 98.80 % | 98.26 % | 97.86 % | 98.85 % |
| PF aligned bases | 99,999,794,784 | 85,048,037,643 | 55,760,979,400 | 87,969,023,949 | 88,331,339,665 |
| PF HQ aligned Q20 bases | 92,108,180,853 | 78,095,506,892 | 51,324,169,931 | 80,781,539,017 | 81,136,683,625 |
| Mean read length | 126 | 126 | 126 | 126 | 126 |
| Reads aligned in pairs | 791,334,073 | 672,294,571 | 439,949,821 | 693,248,273 | 698,741,856 |
| % of reads aligned in pairs | 99.34 % | 99.17 % | 98.94 % | 98.79 % | 99.28 % |
