# Supplementary material for: Genomic Characteristics of Genetic Creutzfeldt-Jakob Disease Patients with V180I Mutation and Associations with Other Neurodegenerative Disorders
Source: PLoS One. 2016 Jun 24;11(6):e0157540. doi: 10.1371/journal.pone.0157540 (PMC4920420; doi:10.1371/journal.pone.0157540)
Supplement: S5 Table — (DOCX) [file pone.0157540.s007.docx]

S5 Table. Primer sequences for variants validation

| **Chr.** | **bp** | **Ref/Alt** | **Gene** | **rs number** | **Primer sequence** | | **Tm (℃)** |
| --- | --- | --- | --- | --- | --- | --- | --- |
| 1 | 89657064 | C/A | GBP4 | - | Forward | CAGGAAACAGCTATGACCGATTTTCCATGCCTTCATT | 60 |
|  |  |  |  |  | Reverse | TGTAAAACGACGGCCAGTTGTGCAATGAACATCTCCT |  |
| 9 | 72912888 | G/T | SMC5 | - | Forward | CAGGAAACAGCTATGACCAAATCCGTTGGGCTTTTA | 60 |
|  |  |  |  |  | Reverse | TGTAAAACGACGGCCAGTCAATTATTTCGCCTTCACA |  |
| 12 | 40677699 | C/T | LRRK2 | rs34410987 | Forward | CAGGAAACAGCTATGACCgtggagctttgtctccata | 60 |
|  |  |  |  |  | Reverse | TGTAAAACGACGGCCAGTAATAAAGGACCAAGCCAAG |  |
